# Identification of Differentially Methylated Sites with Weak Methylation Effects

**DOI:** 10.3390/genes9020075

**Published:** 2018-02-08

**Authors:** Hong Tran, Hongxiao Zhu, Xiaowei Wu, Gunjune Kim, Christopher R. Clarke, Hailey Larose, David C. Haak, Shawn D. Askew, Jacob N. Barney, James H. Westwood, Liqing Zhang

**Affiliations:** 1 Department of Computer Science, Virginia Tech, Blacksburg, VA 24061, USA; hongt1@vt.edu; 2 Department of Statistics, Virginia Tech, Blacksburg, VA 24061, USA; hongxiao@vt.edu (H.Z.); xwwu@vt.edu (X.W.); 3 Department of Plant Pathology, Physiology and Weed Science, Virginia Tech, Blacksburg, VA 24061, USA; gunjunekim@gmail.com (G.K.); hlarose@vt.edu (H.L.); dhaak@vt.edu (D.C.H.); saskew@vt.edu (S.D.A.); jnbarney@vt.edu (J.N.B.); westwood@vt.edu (J.H.W.); 4 Genetic Improvement of Fruits and Vegetables Laboratory, United States Department of Agriculture, Agricultural Research Service, Beltsville, MD 20705, USA; thechrisclarke@gmail.com

**Keywords:** differentially methylated regions, wavelet-based functional mixed model, weak methylation effect

## Abstract

Deoxyribonucleic acid (DNA) methylation is an epigenetic alteration crucial for regulating stress responses. Identifying large-scale DNA methylation at single nucleotide resolution is made possible by whole genome bisulfite sequencing. An essential task following the generation of bisulfite sequencing data is to detect differentially methylated cytosines (DMCs) among treatments. Most statistical methods for DMC detection do not consider the dependency of methylation patterns across the genome, thus possibly inflating type I error. Furthermore, small sample sizes and weak methylation effects among different phenotype categories make it difficult for these statistical methods to accurately detect DMCs. To address these issues, the wavelet-based functional mixed model (WFMM) was introduced to detect DMCs. To further examine the performance of WFMM in detecting weak differential methylation events, we used both simulated and empirical data and compare WFMM performance to a popular DMC detection tool methylKit. Analyses of simulated data that replicated the effects of the herbicide glyphosate on DNA methylation in *Arabidopsis thaliana* show that WFMM results in higher sensitivity and specificity in detecting DMCs compared to methylKit, especially when the methylation differences among phenotype groups are small. Moreover, the performance of WFMM is robust with respect to small sample sizes, making it particularly attractive considering the current high costs of bisulfite sequencing. Analysis of empirical *Arabidopsis thaliana* data under varying glyphosate dosages, and the analysis of monozygotic (MZ) twins who have different pain sensitivities—both datasets have weak methylation effects of <1%—show that WFMM can identify more relevant DMCs related to the phenotype of interest than methylKit. Differentially methylated regions (DMRs) are genomic regions with different DNA methylation status across biological samples. DMRs and DMCs are essentially the same concepts, with the only difference being how methylation information across the genome is summarized. If methylation levels are determined by grouping neighboring cytosine sites, then they are DMRs; if methylation levels are calculated based on single cytosines, they are DMCs.

## 1. Introduction

Deoxyribonucleic acid (DNA) methylation is an important epigenetic mechanism in controlling gene expression, silencing of genes on the inactive X chromosome, imprinted genes, and parasitic DNAs [1]. Accurate characterization of DNA methylation is essential for understanding genotype–phenotype association, gene–environment interaction, diseases, and stress responses [2]. Genome-wide bisulfite-treated DNA sequencing has enabled the measurement of DNA methylation at the single nucleotide resolution. After DNA is treated with sodium bisulfite, unmethylated cytosines (Cs) are converted to uracils, which appear as thymines (Ts) in the output data, whereas methylated Cs remain unchanged. At a single cytosine site, methylation levels are estimated by taking the ratio of C/(T + C), where C and T are the counts of cytosines and thymines, respectively, from all aligned reads at the site, assuming that the conversion rate of unmethylated Cs to Ts is 100%. The count of Ts represents the number of unmethylated Cs, and the count of Cs represents the number of methylated Cs. The most common task is to detect differentially methylated cytosine (DMC) sites across different treatment samples (e.g., dosage vs. non-dosage samples and cases vs. controls). Although numerous statistical methods, such as Fisher’s exact test and logistic regression, have been used for the detection of DMCs [3], several challenges remain.

**Figure 1:**
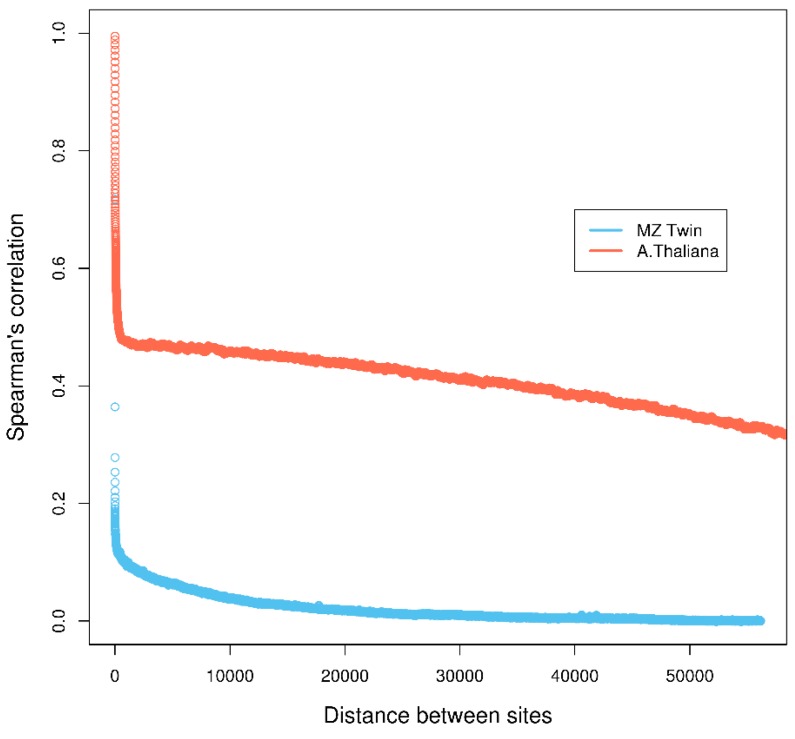
Correlation of methylation levels of neighboring cytosine regions in monozygotic (MZ) twin and neighboring cytosines in *Arabidopsis thaliana* datasets. Details of the calculation are in Section 1 in Supplementary Materials.

First, most methods make the assumption that individual cytosine methylation levels are independent across the genome. This assumption is questionable, as it has been shown that methylation levels of nearby cytosine sites are highly correlated ([4]; Figure 1) and depend on the sequence specificity, i.e., CG, CHG, and CHH (where C=cytosine, G=guanine, H=A (adenine), C or T) in *Arabidopsis thaliana* [5,6], or the nature of the methylated sequence, i.e., transposable elements, repeats exons, introns, promoters, etc. [7]. Assuming independence across cytosine sites can lead to underestimation of p-values and inflated type-I error, resulting in an increased false discovery rate of DMCs [8]. Second, due to the current high cost of whole genome bisulfite sequencing, studies are often done across a small number of biological samples for each phenotype/treatment, which limits statistical power for detecting weak methylation differences. 

To address these issues, Lee and Morris [9] applied the wavelet-based functional mixed model (WFMM) developed in Morris and Carroll [10] to detect DMCs. They examined three human datasets and identified some novel differentially methylated regions that were not detected previously. To further examine the power of WFMM, especially in detecting sites with weak methylation effects, we applied WFMM to existing *Arabidopsis thaliana* data under varying herbicide glyphosate dosages [11] and data from monozygotic (MZ) twins with different pain sensitivities [12]. Both of these datasets were shown to have a small differential methylation effect, i.e., average methylation levels between any two phenotype groups <1%. Here we compare the performance of WFMM with that of the commonly used program methylKit [13] on both empirical and simulated data, and conducted functional analysis for the DMCs identified.

## 2. Methods

### 2.1. Wavelet-Based Functional Mixed Models

Assuming that all methylation measurements come from N individuals across all T genomic locations, a functional mixed effects model can be represented by:

(1) $$y_{i}\left(t\right)=\overset{J+1}{\underset{j=1}{\sum }}X_{ij}B_{j}\left(t\right)+\overset{M}{\underset{m=1}{\sum }}Z_{im}U_{m}\left(t\right)+E_{i}\left(t\right),\;t\in T$$

where *y_i_(t)* represents the logit-transformation of methylation levels at a genomic location *t* *∈* *{t_l_; l = 1, …, T}* for the i-th individual, *i = 1,…,N*. *X_ij_* = 1 if individual i belongs to treatment j and 0 otherwise, for 1 ≤ j ≤ *J*. The function *B_j_(t)* represents the fixed effect corresponding to treatment and other covariates of interest). *Z_im_* a random covariate that takes into account variations in *y_i_(t)* that are caused by potential multilevel structures in the measurements (e.g., when multiple subjects from the same family were measured, then each family will introduce its own random effect and *Z_im_* = 1 if individual i is from family m and *U_m_(t)* is the random effect of family m). *E_i_(t)* is a residual error function. Using vectorized formulation, we may write the model (1) as:

(2) Y(t)=XB(t)+ZU(t)+E(t), t∈T

where ***Y****(t)* = [*Y_1_(t)*,…,*Y_N_(t)*]*^T^*, ***B****(t)* = [*B_1_(t)*,…,*B_J_(t)*]*^T^*, ***U****(t)* = [*U_1_(t*),…,*U_M_(t)*]*^T^* and ***E****(t)* = [*E_1_(t)*,…,*E_N_(t)*]*^T^*. Here, ***Y*** is a *N × T* matrix across all *T* genomic locations for all N individuals. ***X*** is an *N × J* design matrix that indicates which treatment group the N individuals belong to or other covariates of interest (e.g., a phenotype), the **B** *(J × T*) matrix contains the fixed effects of the covariates. The *t*-th column of **B**, denoted by **b***_t_* is a *J*-dimensional vector describing the effects the J covariates on ***Y*** at genomic location *t*.

For example, if we let the *i*-th row of ***X*** be a 1/0 vector to indicate which of the herbicide glyphosate dosage groups the i-th plant was treated, *i = 1,…,N*, then **b***_t_* corresponds to the effect of dose levels on ***Y*** at genomic location *t*. In Equation (2), ***Z*** is a design matrix for random effects that takes into account variations in ***Y*** that are caused by potential multilevel structures in the measurements; ***U*** contains the corresponding random effects; ***E*** is an *N × T* matrix of residual errors. We assume that ***E*** is multivariate normal with mean 0 and variance-covariance matrix S. For example, in our *A. thaliana* experiment, there are four plants for each of the 0%, 5%, and 10% glyphosate-treated groups. Therefore, the ***X*** design matrix is a 12 *×* 3 and **B** is a 3 *× T* matrix, where *T* is the number of cytosine locations. Since the *A. thaliana* data does not involve multilevel structures, the random effect term in Equation (2) is omitted. The resulting functional model can be rewritten as

(3) Y(t)=XB(t)+E(t),t∈T

where

$$X=\left[\begin{array}{c} 1\;\;0\;\;0\\ 1\;\;0\;\;0\\ 1\;\;0\;\;0\\ 1\;\;0\;\;0\\ 0\;\;1\;\;0\\ 0\;\;1\;\;0\\ 0\;\;1\;\;0\\ 0\;\;1\;\;0\\ 0\;\;0\;\;1\\ 0\;\;0\;\;1\\ 0\;\;0\;\;1\\ 0\;\;0\;\;1 \end{array}\right]\;\mathrm{and}\;B=\left[b_{1}\;b_{2}\;b_{3}\ldots b_{T\;}\right].$$

Each **b***_t_* is a column vector consisting of *p* = 3 elements/groups giving the mean methylation profiles for each group at a given genomic location *t*.

To incorporate nearby methylation correlations across all genomic locations *T* into the model, we first use a basis function transform to transform Equation (3) from the original data space into the basis space, and then fit the basis space model to estimate parameters. Finally, we transform results back to the original data space for inference. In particular, we apply the discrete wavelet transform (DWT) to each row of ***Y*** to obtain a N × T* matrix of wavelet coefficients ***D***. The corresponding wavelet space model can be obtained by post-multiplying both sides of Equation (3) by ***Φ’*** the wavelet transformation operator:

(4) $$Y\Phi^{\prime }=XB\Phi^{\prime }+E\Phi^{\prime }$$

**D** = XB* + E* (5)

where ***Φ’*** is a T × T* wavelet transformation operator, ***D*** = ***YΦ’***, B* = **B*Φ’***, and E* = ***EΦ’***. Equation (5) is a wavelet space model with ***D***, B*, and E* representing the wavelet coefficients of ***Y***, ***B***, and ***E***, respectively. We adopt a Bayesian approach to fit Equation (5) following Morris and Carroll [10]. The posterior samples of the parameters in Equation (5) are obtained by employing a Markov chain Monte Carlo (MCMC) algorithm. Inverse DWT is finally applied to the posterior samples of B* to obtain posteriors for ***B*** in the data domain, which were subsequently used to identify DMCs following a Bayesian false discovery rate approach.

### 2.2. Bayesian False Discovery Rate 

Based on the posterior samples of ***B***, we can identify significant regions either on ***B*** or on the contrast effects that contains the differences between covariate effects in ***B***. For example, in the *A. thaliana* data example, since we are interested in identifying DMCs with different dosage effects, we will calculate the contrast effects by pre-multiplying ***B*** with a contrast effect operator $\left(\begin{array}{ccc} -1 & 1 & 0\\ 0 & -1 & 1\\ -1 & 0 & 1 \end{array}\right)$, which transforms the effect of each dosage level to the contrast effects of Level 2 vs. Level 1, Level 3 vs. Level 2, and Level 3 vs. Level 1, respectively. We will apply this operator to all posterior samples of B to obtain the posterior samples of the contrast effects. Denote C_α_(t), *t* *∈* *{t_l_; l = 1, …, T}* the α th contrast effect, identifying significant DMCs on C_α_(t) amounts to identifying locations on $C_{a}\left(t\right)$ that are large in magnitude. We achieve this by performing a Bayesian multiple testing that controls the overall false discovery rate following Morris et al. [10], Zhu et al. [14], and Lee and Morris [9].

Specifically, in the Bayesian FDR approach, we detect locations in *t* *∈* *{t_l_; l = 1, …, T}* that has C_α_(t) values greater than some threshold δ (in absolute value) based on G posterior samples of C_α_(t) for all contrast effects. We first calculate the pointwise posterior probability of at least δ difference at *t_l_* by calculating ${\hat{p}}_{a}\left(t_{l}\right)=\mathrm{Pr}\left\{\left|C_{a}\left(t_{l}\right)\right|>\delta |Y\right\}\approx \frac{\sum_{g=1}^{G}I\{|C_{a}{\left(t_{l}\right)}^{\left(g\right)}|>\delta \}}{G}$, where C_α_(t)^(*g*)^ denotes the *g*-th sample of C_α_(t) at *t_l_*. Then, we find a cut-point *ϕ_α_* for ${\hat{p}}_{a}$*(t_l_)* so that the expected global Bayesian FDR is less than or equal to a pre-specified level α. We claim all of the *t_l_* on which ${\hat{p}}_{a}$*(t_l_)* >*ϕ_α_* as genomic locations with C_α_(*t_l_*) greater than δ.

## 3. Data and Simulation

### 3.1. Arabisopsis thaliana Treated with Herbicide Glyphosate

We previously investigated methylation profiles of twelve *A. thaliana* plants exposed to the herbicide glyphosate at different dosage concentrations [11]. In these experiments, blocks of four *A. thaliana* plants were randomly assigned to glyphosate treatment at three different dosages, 0% (control), 5%, and 10% of the label recommended field application rate. We intended to impose stress while still allowing the plants to survive and reproduce. Following glyphosate treatment, these plants were transferred to a growth chamber with a 12 h light cycle and a light intensity of 90 µmol m^−2^ s^−1^ and allowed to grow for approximately 2 weeks for the 0% and 5% glyphosate-treated plants and 8 weeks for the 10% glyphosate-treated plants until fully developed siliques were formed. Following 0, 5 and 10% glyphosate exposure on four-week-old rosettes of the twelve *A. thaliana* individuals, genomic DNA were isolated from cauline leaves of the newly matured siliques using Biosprint-15 plant DNA extraction kit (Qiagen, Hilden, Germany). The tissue samples from these 12 plants were sent to the Genomics Research Laboratory at the Biocomplexity Institute of Virginia Tech for sequencing. One hundred nanograms of DNA samples were bisulfite converted using EZ DNA methylation-Gold Kit (#D5005, Zymo Research, Irvine, CA, USA). Illumina DNA libraries were prepared from the above purified bisulfite converted DNA samples using EpiGnome Methyl-Seq kit (Epicentre, Illumina Inc., Madison, WI, USA). In the end, each of six samples were barcoded, quantified by qPCR, and pooled to sequence on Illumina Hiseq Rapid Run flowcell (Illumina, San Diego, CA, USA). The bisulfite short reads dataset can be download from NCBI Sequence Read Archive (SRA) BioProject ID: PRJNA322493. In total, there were 872,608,912 bisulfite paired-end short reads with a length of 100 bp for each end. The coverage depth ranged from 48.6 to 76.3× across all samples. First, the sequenced reads’ quality was checked using FastQC [15] to eliminate adapter sequences and barcodes using Trimmomatic [16] and FastX Tookit [17]. Low-quality reads (quality score Q < 30) were excluded. After all quality checks, bisulfite short sequences were aligned to the *A. thaliana* from Arabidopsis Information Resource version 10 (TAIR 10) reference genome using Bismark aligner (v 0.14.5) with default parameters (n = 1 and l = 50) [18]. Cytosine methylation level information was extracted from aligned reads using Bismark methylation extractor. A total of 3,348,756 cytosines passed the preprocessing steps and thus serve as the basis on which we detect significant methylated cytosines differentiating glyphosate dosage groups.

### 3.2. Methylation Level Simulation

We aimed to generate methylation profiles that closely mimic the real data collected from our experiment [7] (Figure S1). Generating a simulated dataset allowed us to evaluate the impact of different parameters on the performance of WFMM. For comparison purposes, we generated two sets of methylated cytosines, one set with correlation among nearby cytosine sites, and the other without methylation correlation. For the uncorrelated dataset, we first randomly selected 10,000 out of the total 100,000 cytosine sites as DMCs (~10% of all cytosine sites are differentially methylated). For each of the three dosage groups, i.e., no treatment (0%) or two different sub-lethal doses (5% and 10%) of glyphosate, we set the average methylation level according to the estimate from the real *A. thaliana* dataset above. The three pairwise mean methylation differences at each cytosine site: for 0% vs. 5%, 5% vs. 10%, and 0% vs. 10%, were set similarly based on the real *A. thaliana* dataset. If one of the mean methylation differences was greater than 0.04, the corresponding cytosine site was considered differentially methylated. In this way, the differentially methylated sites (true positive methylation differentiation) in simulated data were generated. On the other hand, if none of the mean methylation differences between any of the two groups were greater than 0.04, the corresponding cytosine site was considered non-differential (true negative methylation differentiation).

To generate correlated simulated datasets, we first divided the real *A. thaliana* dataset into blocks of 100,000 cytosine sites and randomly chose blocks to generate methylation profiles for simulated data. These random blocks were to ascertain that the correlation structure in the real *A. thaliana* dataset was maintained in the simulated datasets with correlated methylation sites. For each random block, if one of the mean methylation differences was greater than 0.04, cytosines were considered differentially methylated, so the methylation levels at these cytosine sites were used to generate methylation profiles for differentially methylated sites. Otherwise, sites were considered non-differential and used to simulate true negative methylation profiles (Figure S2). Individual methylation levels for each of the three dosage groups from both correlated and uncorrelated datasets were generated from truncated, normally distributed data that ranged from 0 to 1, with mean and standard deviations calculated from the real *A. thaliana* dataset.

We changed methylation difference profiles by altering the cutoff value for specifying a DMC from 0.04 to 0.08, 0.1, 0.12, 0.15, 0.2, and 0.25. For example, with a cutoff value of 0.25, only cytosines with at least one of the pairwise mean methylation differences greater than 0.25 were considered differentially methylated. We also increased sample sizes for each dosage group from 4 to 10, to 20, to 30, and then to 40 to examine how the WFMM method performs under different scenarios and compared its performance to the commonly used program methylKit [13].

## 4. Results

### 4.1. Simulation Results

#### 4.1.1. Effect of the Degree of Methylation Difference

**Figure 2:**
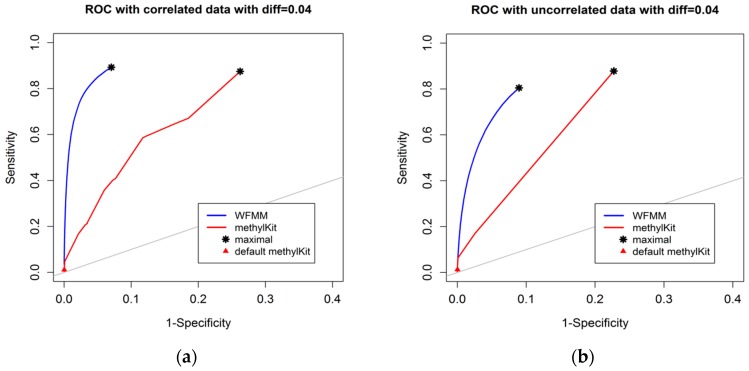

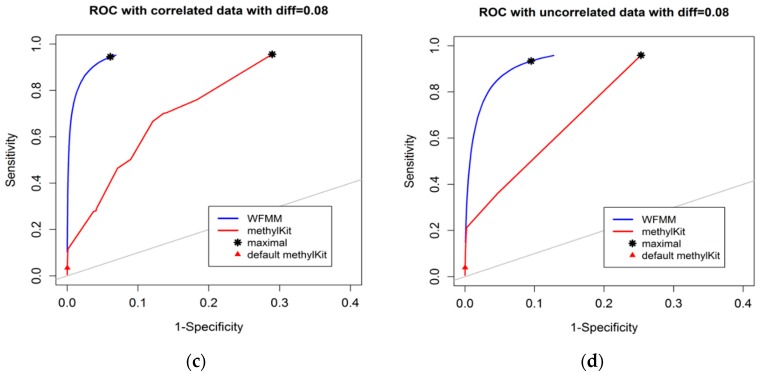
Receiver operating characteristic (ROC) curve comparison between wavelet-based functional mixed model (WFMM) (blue curve) and methylKit (red curve) when the differentially methylated cutoff is 0.04 in correlated cytosines (**a**) and uncorrelated cytosines (**b**) and when the differentially methylated cutoff is 0.08 in correlated cytosines (**c**) and uncorrelated cytosines (**d**). The gray line represents points where sensitivity equals specificity.

The degree of differential methylation between different phenotypes is an obvious factor to consider when examining the performance of tools for detecting differentially methylated cytosines. In our analysis, we calculated the receiver operating characteristic (ROC) curves from the WFMM method and methylKit [13] under different degrees of methylation difference. Figure 2 shows the performance of the two methods with different methylation difference cutoffs. We used Youden’s rule to find the optimal threshold for the delta parameter (δ) in WFMM and the *q* value parameter in methylKit. MethylKit uses *q* value —*p* values adjusted for multiple-testing. According to Youden’s rule, the optimal threshold is where the sum of sensitivity and specificity is maximized. Figure 2 shows that overall WFMM performs better than methylKit with higher sensitivity and specificity in both correlated and uncorrelated scenarios. When the differentially methylated cutoff is 0.04 or 0.08 and in both correlated and uncorrelated cytosines, the optimal value for the δ parameter in WFMM is 0.01 and the optimal value for the *q* value parameter in methylKit is 1.00. We note that there is an improved performance in WFMM, i.e., higher specificity and slightly higher sensitivity when comparing the correlated data with the uncorrelated data, whereas the methylKit performance is similar in both scenarios.

**Figure 3:**
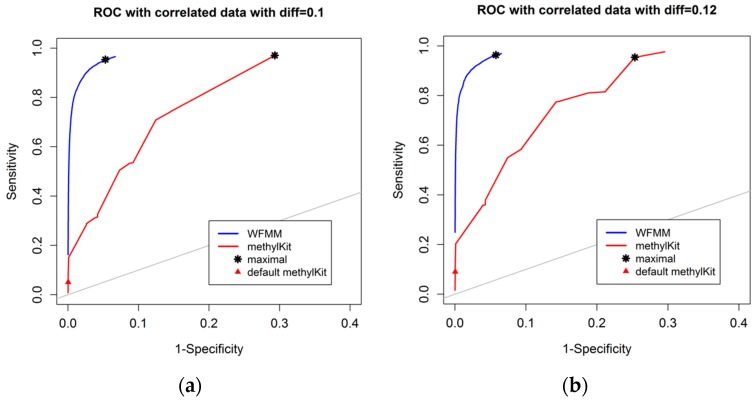

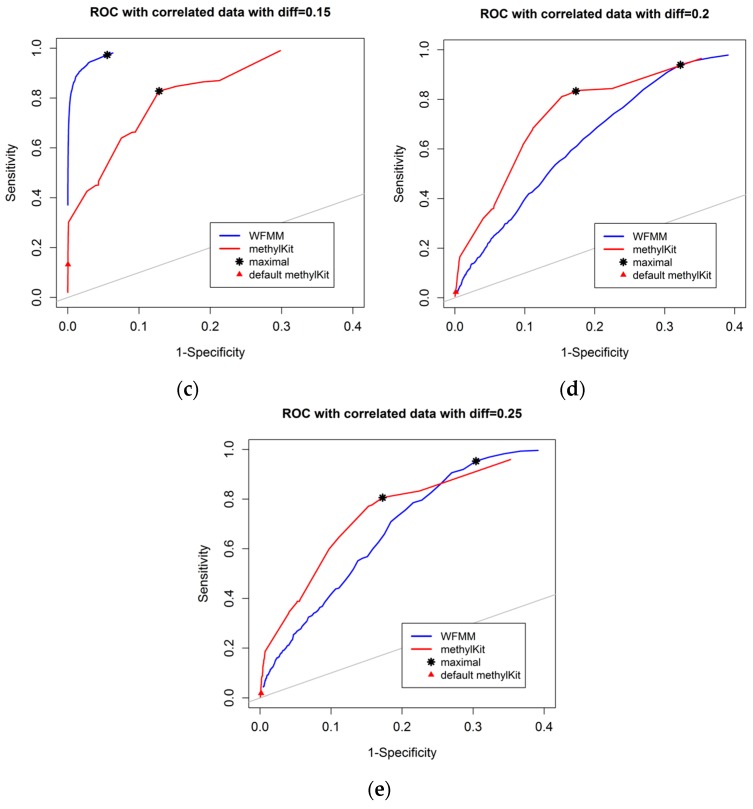
ROC curves of WFMM (blue curve) and methylKit (red curve) as differentially methylated cutoff increases from 0.1, to 0.25 (diff = 0.1, 0.12, 0.15, 0.2, 0.25).

Figure 3 shows that, as the differentially methylated cutoff increases from 0.1 to 0.25, the gaps in the ROC curves between WFMM and methylKit become narrower. Specifically, there is little improvement in WFMM, whereas the performance of methylKit improves with increasing differentially methylated cutoff values. When the differentially methylated cutoff is 0.2 or 0.25, WFMM and methylKit perform similarly. To illustrate, when the differentially methylated cutoff is 0.25, optimal threshold δ = 0.013 in WFMM, and optimal threshold *q* value = 0.76 in methylKit, WFMM, compared with methylKit, has a higher sensitivity (0.953 vs. 0.806) but a lower specificity (0.696 vs. 0.828). Therefore, there is a trade-off between sensitivity and specificity when choosing between the two methods; one method might produce higher sensitivity but lower specificity or vice versa.

#### 4.1.2. Effect of Sample Size

**Figure 4:**
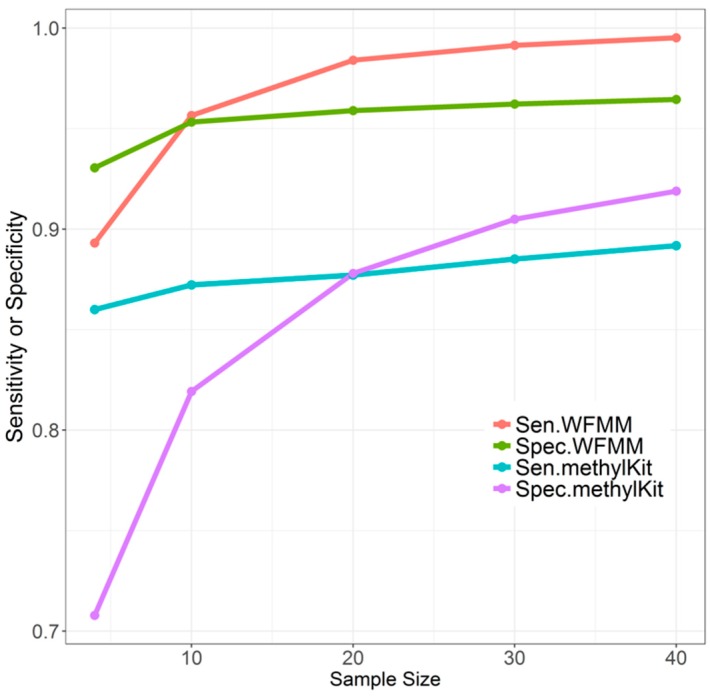
Effect of different sample sizes on WFMM with δ = 0.01 and methylKit with adjusted settings (*q* value = 1.00; difference = 4) using correlated simulated data when the differentially methylated cutoff is 0.04.

Overall, when the sample size increases from 4 to 10, to 20, to 30, and then to 40, WFMM performance remains stable (Figure 4). There is a moderate improvement in sensitivity and specificity when the sample size increases from 4 to 10. There is only slight improvement in sensitivity and specificity when the sample size exceeds 10. In contrast, increasing sample size results in dramatic improvement of the specificity of methylKit, while the sensitivity only improves slightly (Figure 4). Therefore, sample size tends to have a larger effect on methylKit than WFMM. It can be inferred that increased sample sizes give methylKit more power to detect small methylation differences across different phenotype groups, whereas WFMM is robust with respect to sample sizes because this method incorporates methylation levels of nearby cytosines to make inferences rather than solely relying on sample size. 

### 4.2. Real Data from Herbicide Glyphosate Treatment of *Arabidopsis thaliana*

**Figure 5:**
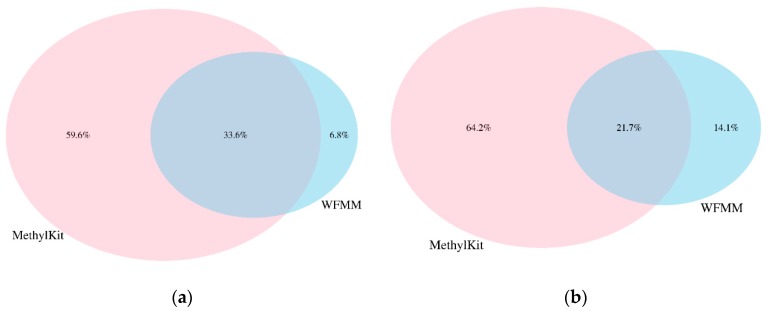
Percentages of overlapping differentially methylated cytosine (DMCs) from methylKit with adjusted settings (difference = 4; *q* value = 1.00) and WFMM with δ = 0.01 in correlated simulated data when the differentially methylated cutoff is 0.04 (**a**) and in the real data (**b**).

We applied WFMM and methylKit on the dataset generated from our herbicide glyphosate treatment experiment on *A. thaliana* [11]. WFMM was able to detect 557,664 DMCs (~17% of all cytosines in the *A. thaliana* genome) corresponding to 15,823 TAIR genes recognized from Database for Annotation, Visualization and Integrated Discovery (DAVID) [19]. In contrast, methylKit detected only 48,041 DMCs (~1.43% of all cytosines in the *A. thaliana* genome) corresponding to 12,166 TAIR genes with default settings (*q* value= 0.01 and difference = 25). When settings were adjusted (*q* value = 1.00 and difference = 4), methylKit detected 1,338,219 DMCs (~40% of all cytosines in the *A. thaliana* genome) corresponding to 30,947 TAIR genes. Table 1 shows the breakdown of the number of significant DMCs and TAIR genes for each chromosome in the *A. thaliana* genome. Chromosomes 1 and 5 have the highest number of genes responding to herbicide glyphosate stress. Analysis of the overlapping DMCs between WFMM and methylKit shows that there are 33.6% and 21.7% common DMCs detected by both WFMM and methylKit in simulated and real datasets, respectively (Figure 5).

**Figure 6:**
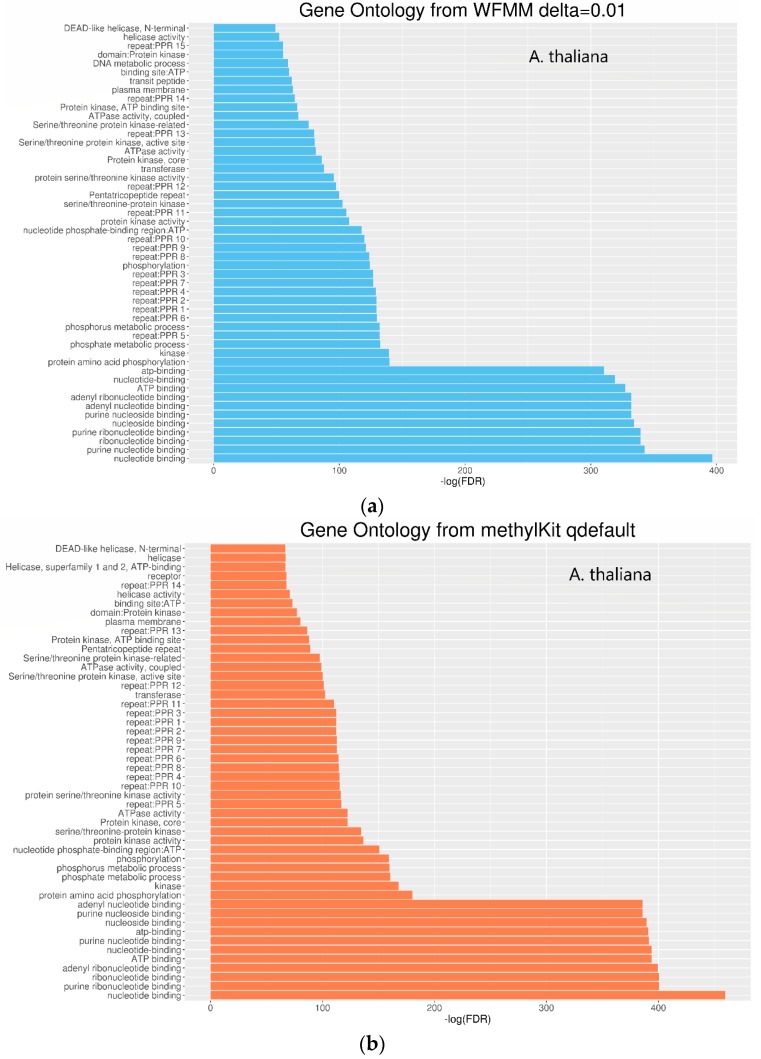
Gene ontology of molecular function for significant differentially methylated TAIR genes detected by WFMM with δ = 0.01 (**a**) and methylKit with default settings (difference = 25; *q* value = 0.01) (**b**).

**Figure 7:**
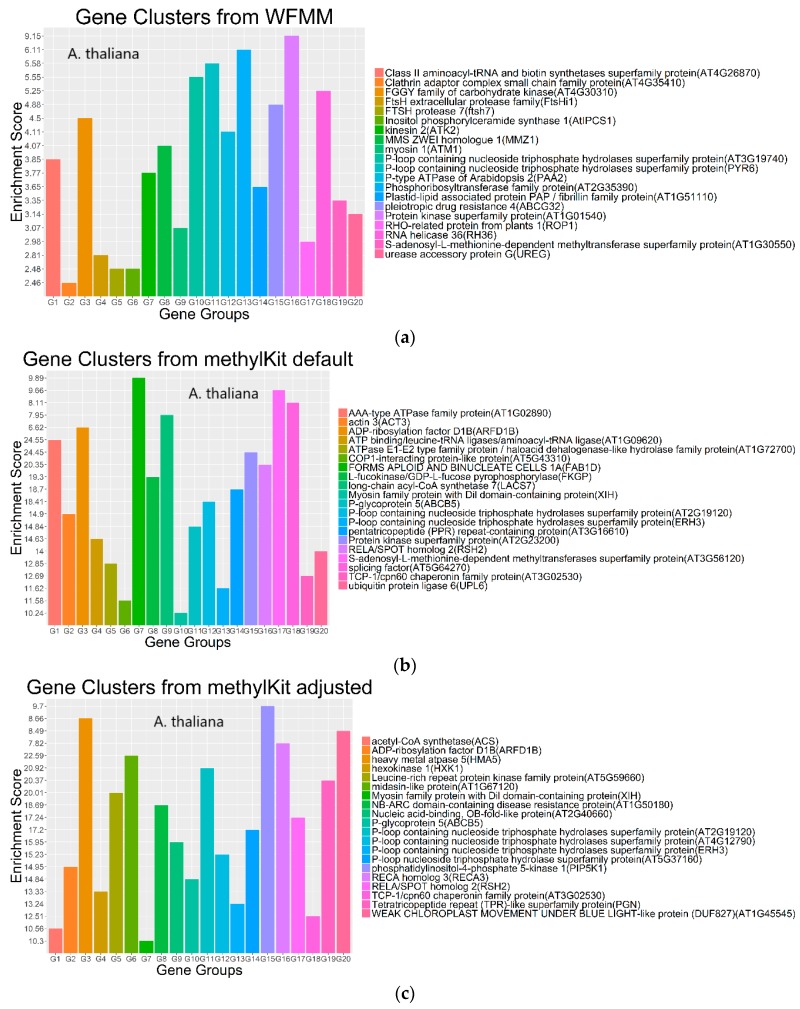
Gene clusters based on the gene ontology of molecular function for the top 3000 most significant genes from WFMM with δ = 0.01 (**a**), methylKit with default settings (difference = 25; *q* value = 0.01) (**b**), and methylKit with adjusted settings (difference = 4; *q* value= 1.00) (**c**).

Functional annotation of the significant genes detected by WFMM and methylKit show similar results between both methods (Figure 6). The most significant gene ontology (GO) terms in WFMM are also found in the top 50 significant methylKit GO terms. Das et al. [20] conducted a similar experiment by applying glyphosate to *A. thaliana* plants and identified 484 genes that might be responsive to glyphosate stress. Comparatively, methylKit with default settings identified 12,166 genes, 181 of which overlap with Das et al. [16], and with adjusted settings (difference = 4; *q* value= 1.00), identified 30,947 genes (mostly *A. thaliana* genes), 466 of which overlap with those identified in Das et al. [16]. In contrast, WFMM with δ = 0.01 identified 12,166 genes, 238 of which overlap with those previously identified [16] (Table 2). Thus, untuned, WFMM is slightly better than methylKit, as it identifies genes slightly more related to glyphosate responses. For a fair comparison, of the 3000 most significant genes, methylKit with default settings has 39 genes, while methylKit with relaxed settings (difference = 4; *q* value = 1.00) has 41 overlapped genes. WFMM with default setting δ = 0.01 has 51 overlapped genes (Table 2). Though there are minor differences in gene clusters between methylKit and WFMM with δ = 0.01, the GO analysis results from the two methods are very similar (Figure 6 and Figure 7).

### 4.3. Real Data from Monozygotic Twin Data with Different Pain Sensitivity Scores

We used the methylation profiles of 25 MZ twin pairs (50 MZ twins) who were discordant for heat pain sensitivity, for model comparison. Datasets were downloaded from Bell et al. [12] with sample IDs from GSM1278649 to GSM1278698. This 25 twin pair dataset was from the discovery phase of Bell et al.’s experiment [12] and is only the first part of their dataset. Heat pain tolerance between twins was determined experimentally using quantitative sensory testing. Whole-blood DNA were assayed using DNA immunoprecipitation, followed by deep sequencing methylated DNA immunoprecipitation (MeDIP-seq). Assay validation, bisulfite conversion, and pyrosequencing were performed by EpigenDx. On average, there were 50 million paired-end reads with a length of 50 bp for each end per individual. These reads were aligned to reference genome hg18 using MAQ (v0.7.1) [21] with default settings. Post-quality control was performed to ensure high quality alignment for methylation quantification [12]. The methylation levels in these datasets were summarized by combining cytosine regions rather than single cytosine sites. In total, there are 5,735,431 DMRs in these datasets. We assigned MZ twins in each of the 25 MZ pairs to two groups according to MZ twins’ pain sensitivity temperatures (high or low). For example, for a MZ twin pair from Family ID 1, MZ Twin 1 and MZ Twin 2 have pain sensitivity temperatures of 44.7 °C and 47.8 °C, respectively. Therefore, we assigned MZ Twin 1 to the low pain sensitivity temperature group and MZ Twin 2 to the high pain sensitivity temperature group. 

**Figure 8:**
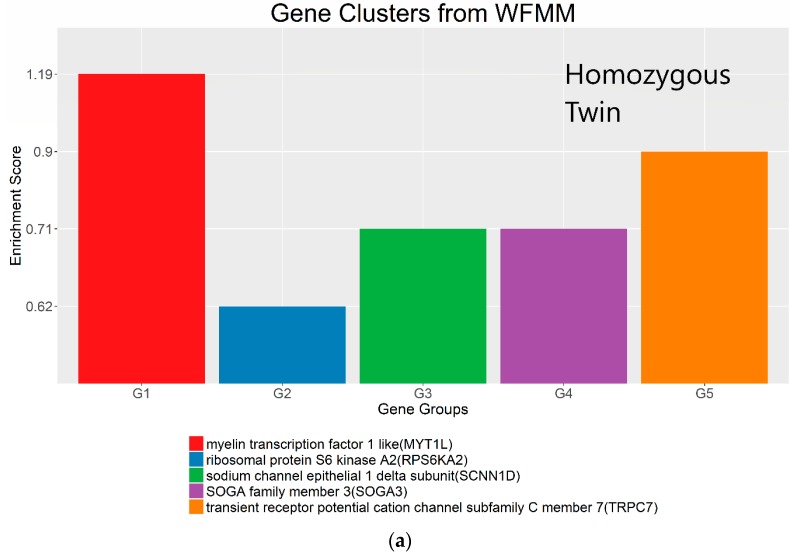

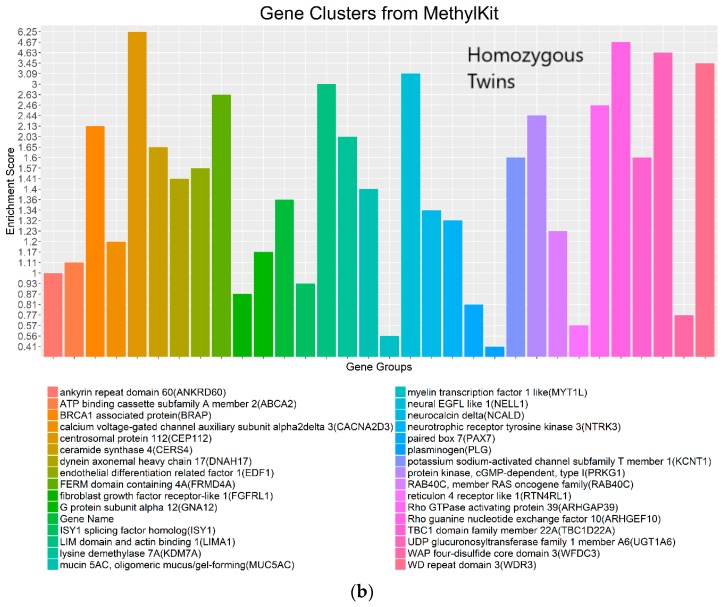
Gene clusters based on the gene ontology of molecular function for significant genes detected by WFMM with δ = 3.44 × 10^−5^ (**a**) and methylKit (difference = 4.34 × 10^−5^; *q* value = 1.00) (**b**).

The WFMM and methylKit were applied to the 50 MZ twins’ methylation profiles with high vs. low pain sensitivity temperatures as phenotypic groups. There were no significant DMRs detected by WFMM with δ = 0.01, methylKit with default settings, or methylKit adjusted settings (difference = 0.04; *q* value= 1.00). This may be because the mean methylation differences between high vs. low pain temperature groups are very small (~4.1% of all mean methylation differences across DMRs <10^−5^) (Figure S3). Therefore, we adjusted parameter settings in both WFMM with δ = 3.44 × 10^−5^ and methylKit (difference = 4.34 × 10^−5^; *q* value= 1.00). These parameter settings from both methods were determined by an empirical function applied on the real twin data and is further described in Section 5. For the 769 significant DMRs detected by WFMM with δ = 3.44 × 10^−5^, there were 236 genes recognized by the gene function enrichment program DAVID (Table 3). These genes were clustered into five groups by DAVID (Figure 8; top panel). For the 2023 significant DMRs from methylKit (difference = 4.34 × 10^−5^; *q* value= 1.00), there were 892 genes recognized by DAVID (Table 3) that were clustered into 32 clusters (Figure 8; bottom panel).

The most important gene groups were ranked by the enrichment scores (EASE scores). The EASE scores are calculated from the geometric mean of all enrichment *p* values for each annotation term of all gene members in a gene group [22]. Two gene clusters that have the highest EASE scores from significant differentially methylated genes detected by WFMM contain myelin transcription factor 1-like (MYT1L, enrichment score = 1.19) and transient receptor potential cation channel subfamily C member 1 (TRPC7, enrichment score = 0.90). MYT1L functions in the developing mammalian central nervous system. TRPC7 was identified by Bell et al. [12] responsive to heat pain sensitivity. In comparison, methylKit was not able to capture relevant gene clusters pertaining to pain sensitivity in its first top 17 clusters. In the 18th cluster, two genes (out of the 112 genes in this cluster) ST6GALNAC1 and TRPC7 were found involved in heat pain sensitivity by Bell et al. [12]. It is remarkable that WFMM was able to capture the significant gene groups related to pain sensitivity using only the 25 MZ twin pairs’ methylation profiles whose methylation differences are very small, whereas Bell et al. [12] had to use the methylation profiles of 25 MZ twin pairs together with 50 unrelated individuals in a meta-analysis to capture the genes responsible for heat pain sensitivity.

## 5. Discussion

Though there are many statistical methods for detecting differentially methylated cytosines, small sample sizes and small differences in methylation data across phenotype groups remain a challenge for these methods [9]. Our analyses demonstrated that the wavelet-based functional mixed model has several advantages over the current standard methylKit. 

First, simulation results show that the WFMM method is robust with respect to small sample sizes (Figure 3). Second, the method is particularly effective for cases where methylation differences across phenotype groups are relatively small. For example, as demonstrated in our MZ twin pair analysis (Figure 8), WFMM can capture significant regions that are relevant to the phenotype of interest. Third, WFMM is able to correct for methylation correlation in the data and therein has improved power in detecting DMCs/DMRs, as illustrated in the *A. thaliana* and MZ twin data analyses. Finally, our analyses revealed that using the default settings of the DMR analysis tools may not be suitable for some types of biological data, as shown in the *Arabidopsis* and twin datasets. We recommend some empirical rules to adjust the default settings so that the method can be better adapted to different methylation profiles of real datasets. For methylKit, we suggest setting the “diff” parameter to be at the 100(1 − E)th quantile of the absolute pairwise methylation level differences between two phenotype groups across the whole genome, where E is an expected percentage of methylation differences across all cytosines for a particular dataset based on prior knowledge. For example, in our *Arabidopsis* data, we expect ~10% (E = 10%) of cytosines to be DMCs. Therefore, we set diff = 0.04 (corresponding to the 90th quantile of the absolute pairwise methylation level differences between phenotype categories). In the twin dataset, we expect E = 0.3%; therefore, we adjust diff in methylKit to 4.34 × 10^−5^ (i.e., the 99.7th quantile of the absolute pairwise methylation level differences across whole human genome). In methylKit, the *q* value parameter should also be adjusted accordingly. If diff is very small (<0.1), set *q* value = 1.00 to collect all significant DMRs. Similarly, WFMM can be empirically tailored to different methylation profiles by controlling the δ parameter, setting δ to be the difference between the 100(1 − E)th quantile of the absolute pairwise methylation differences between two phenotype groups across the whole genome and the standard deviation of the methylation differences. For example, in our *A. thaliana* dataset, the 90th quantile of the absolute pairwise methylation level differences between dosage categories is 0.04 and the standard deviation of pairwise methylation level differences between phenotype categories is 0.03; therefore, δ = 0.04 − 0.03 = 0.01. In the twin dataset, the corresponding 99.7th quantile and standard deviation are 4.34 × 10^−5^ and 9.2 × 10^−6^, respectively; therefore, we use δ = 4.34 × 10^−5^ − 9.2 × 10^−6^ = 3.44 × 10^−5^. In this way, a better DMC detection result can be achieved based on different methylation datasets.

## Figures and Tables

**Table 1:** The number of significant differentially methylated cytosine (DMCs), and genes recognized by Database for Annotation, Visualization and Integrated Discovery (DAVID) by applying wavelet-based functional mixed model (WFMM) with δ = 0.01 and methylKit with default settings (difference = 25; *q* value= 0.01) and methylKit with adjusted settings (difference = 4; *q* value= 1.00) on a real *A. thaliana* dataset.

Chromosome	WFMM δ = 0.01; Number of DMCs	methylKit Default; *q* value = 0.01; Difference = 25, Number of DMCs	methylKit *q* value = 1.00; Difference = 4, Number of DMCs	WFMM δ = 0.01; Number of Significant Genes	methylKit Default; *q* value = 0.01; Difference = 25; Number of Significant Genes	methylKit *q* value = 1.00; Difference = 4; Number of Significant Genes
Chr1	133,512	12,048	294,153	4041	3098	7760
Chr2	87,488	7627	244,683	2417	1887	5129
Chr3	113,229	9863	274,382	3180	2459	6254
Chr4	91,327	7708	227,539	2563	1943	4815
Chr5	123,027	10,776	290,090	3622	2779	6989
ChrC *	9081	19	7306	0	0	0
ChrM *	0	0	66	0	0	0
Total	557,664	48,041	1,338,219	15,823	12,166	30,947

* ChrC stands for chloroplast; ChrM designates mitochondria.

**Table 2:** Number of intersecting genes between 484 genes identified by Malay Das et al. [20] that are related to herbicide glyphosate stress and significant genes identified by WFMM and methylKit.

Methods	Number of Significant DMRs	Number of Significant Genes Using DAVID
WFMM δ = 3.44 × 10^−5^	769	236
methylKit adjusted; *q* value = 1.00; difference = 4.34 × 10^−5^	2023	892

**Table 3:** Number of significant DMCs, and genes recognized by DAVID by applying WFMM with δ = 3.44 × 10^−5^ and difference = 4.34 × 10^−5^; *q* value= 1.00 on 25 monozygotic (MZ) twin pairs with different pain sensitivity temperature.

Methods	Number of Significant Genes	Number of Shared Genes in All Significant Genes	Number of Shared Genes in Top 3000 Most Significant Genes
WFMM δ = 0.01	15,823	238	51
methylKit default; *q* value = 0.01; difference = 25	12,166	181	39
methylKit adjusted; *q* value = 1.00; difference = 4	30,947	466	44

## Supplementary Materials

The following are available online at http://www.mdpi.com/2073-4425/9/2/75/s1. Section 1: Calculation of correlations of methylation levels between any cytosine site with its neighboring cytosine; Figure S1: Pairwise mean methylation Difference Profile of 12 *A. Thaliana* plants after glyphosate treatment; Figure S2: Methylation level simulation at cytosine sites. Uncorrelated methylated cytosine simulated data (left panel) and correlated methylated cytosine simulated data (right panel); Figure S3: Mean methylation profiles between higher and lower pain temperature group in 25 MZ twin pairs; Table S1: Number of significant DMCs, genes recognized by Ensemble by applying WFMM with δ = 4 × 10^−5^ and *q* value = 1.01, difference = 0.07, on 25 monozygotic twin pairs with different pain sensitivity temperatures for each chromosome.
