# Supplementary material for: Identification of Differentially Methylated Sites with Weak Methylation Effects
Source: Genes (Basel). 2018 Feb 8;9(2):75. doi: 10.3390/genes9020075 (PMC5852571; doi:10.3390/genes9020075)
Supplement: Supplementary file 1 [file genes-09-00075-s001.pdf]

SUPPLEMENTARY

Section 1: Calculation of correlations of methylation levels between any cytosine site with its neighboring cytosine sites

Suppose we have four *A. thaliana* replicates for each of the three dosage level herbicide as in our glyphosate herbicidal experiment. At a given cytosine *i*, methylation levels for each replicate at each dosage level are

0% dosage glyphosate from four *A. thaliana* replicates: $a_{1i}, a_{2i}, a_{3i}, a_{4i}$

5% dosage glyphosate from four *A. thaliana* replicates: $b_{1i}, b_{2i}, b_{3i}, b_{4i}$

10% dosage glyphosate from four *A. thaliana* replicates: $c_{1i}, c_{2i}, c_{3i}, c_{4i}$

We pool all samples across all cytosine sites and neighboring cytosines of distance =1 into two vectors

v_1=$(a_{11}, a_{21}, a_{31}, a_{41}, \dots, a_{1i}, a_{2i}, a_{3i}, a_{4i}, b_{11}, b_{21}, b_{31}, b_{41}, \dots, b_{1i}, b_{2i}, b_{3i}, b_{4i}, c_{11}, c_{21}, c_{31}, c_{41}, \dots, c_{1i}, c_{2i}, c_{3i})$

v_2=$(a_{21}, a_{31}, a_{41}, \dots, a_{1i}, a_{2i}, a_{3i}, a_{4i}, b_{11}, b_{21}, b_{31}, b_{41}, \dots, b_{1i}, b_{2i}, b_{3i}, b_{4i}, c_{11}, c_{21}, c_{31}, c_{41}, \dots, c_{1i}, c_{2i}, c_{3i}, c_{4i})$

We then calculate Spearman's correlation ρ_1 of v_1 and v_2

Similarly, we can calculate Spearman's correlation ρ_2 of v_1 and v_2 from pooling all samples across all cytosine sites and neighboring cytosines of distance =2 into two vectors

v_1=$(a_{11}, a_{21}, a_{31}, a_{41}, \dots, a_{1i}, a_{2i}, a_{3i}, a_{4i}, b_{11}, b_{21}, b_{31}, b_{41}, \dots, b_{1i}, b_{2i}, b_{3i}, b_{4i}, c_{11}, c_{21}, c_{31}, c_{41}, \dots, c_{1i}, c_{2i})$

v_2=$(a_{31}, a_{41}, \dots, a_{1i}, a_{2i}, a_{3i}, a_{4i}, b_{11}, b_{21}, b_{31}, b_{41}, \dots, b_{1i}, b_{2i}, b_{3i}, b_{4i}, c_{11}, c_{21}, c_{31}, c_{41}, \dots, c_{1i}, c_{2i}, c_{3i})$

Similar Spearman's correlation calculation is done on monozygotic twins. All $\rho_1, \rho_2, \dots, \rho_n$ are plotted against distance between cytosine sites of *1, 2, …, n* from real *A. thaliana* (red curve) and monozygotic twins (blue curve) to create Fig 1.

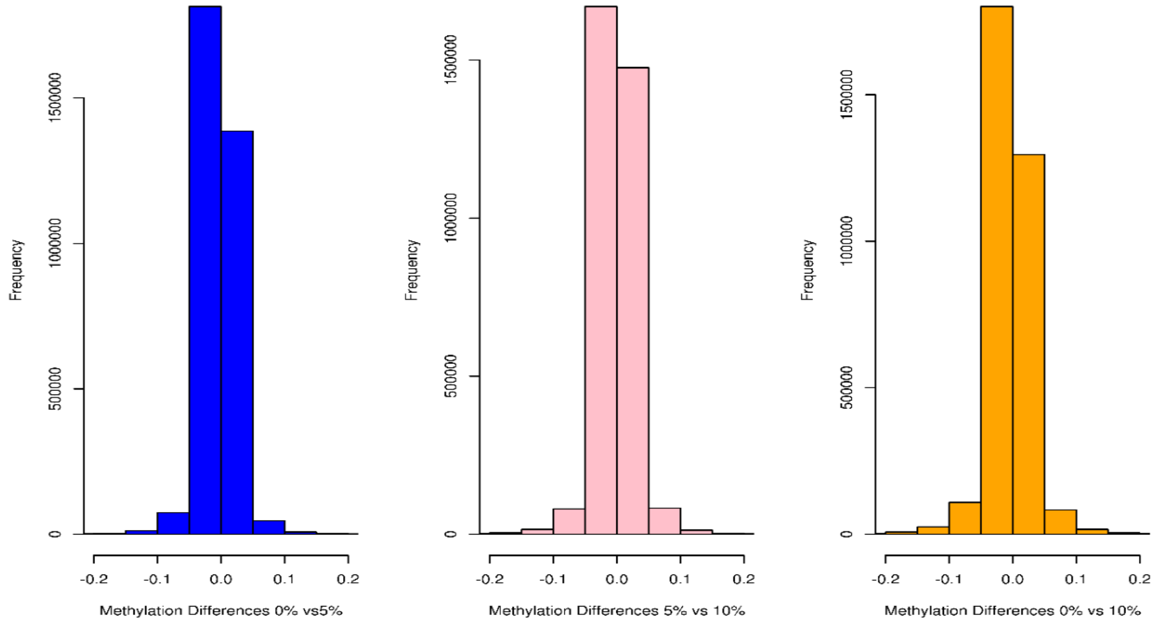

Figure S1: Pairwise mean methylation Difference Profile of 12 *A. Thaliana* plants after glyphosate treatment

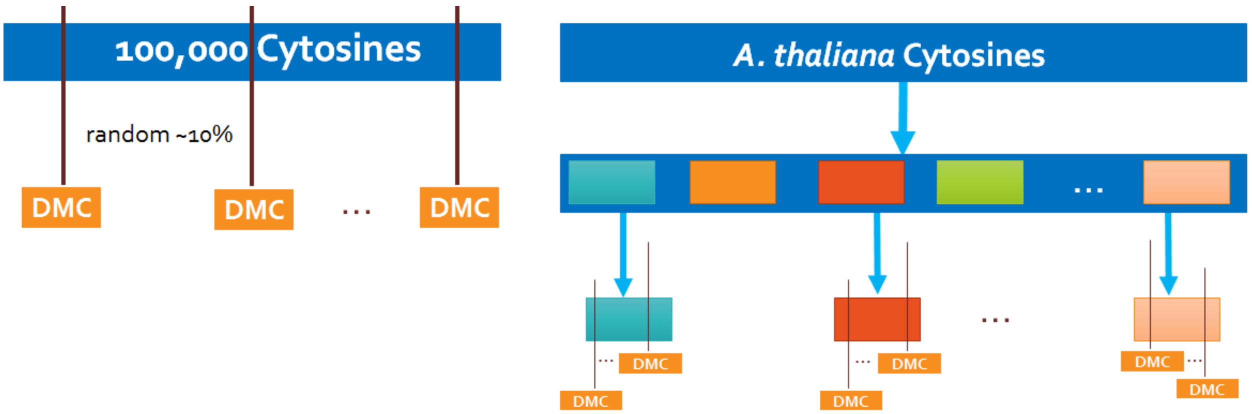

Figure S2: Methylation level simulation at cytosine sites. Uncorrelated methylated cytosine simulated data (left panel) and correlated methylated cytosine simulated data (right panel)

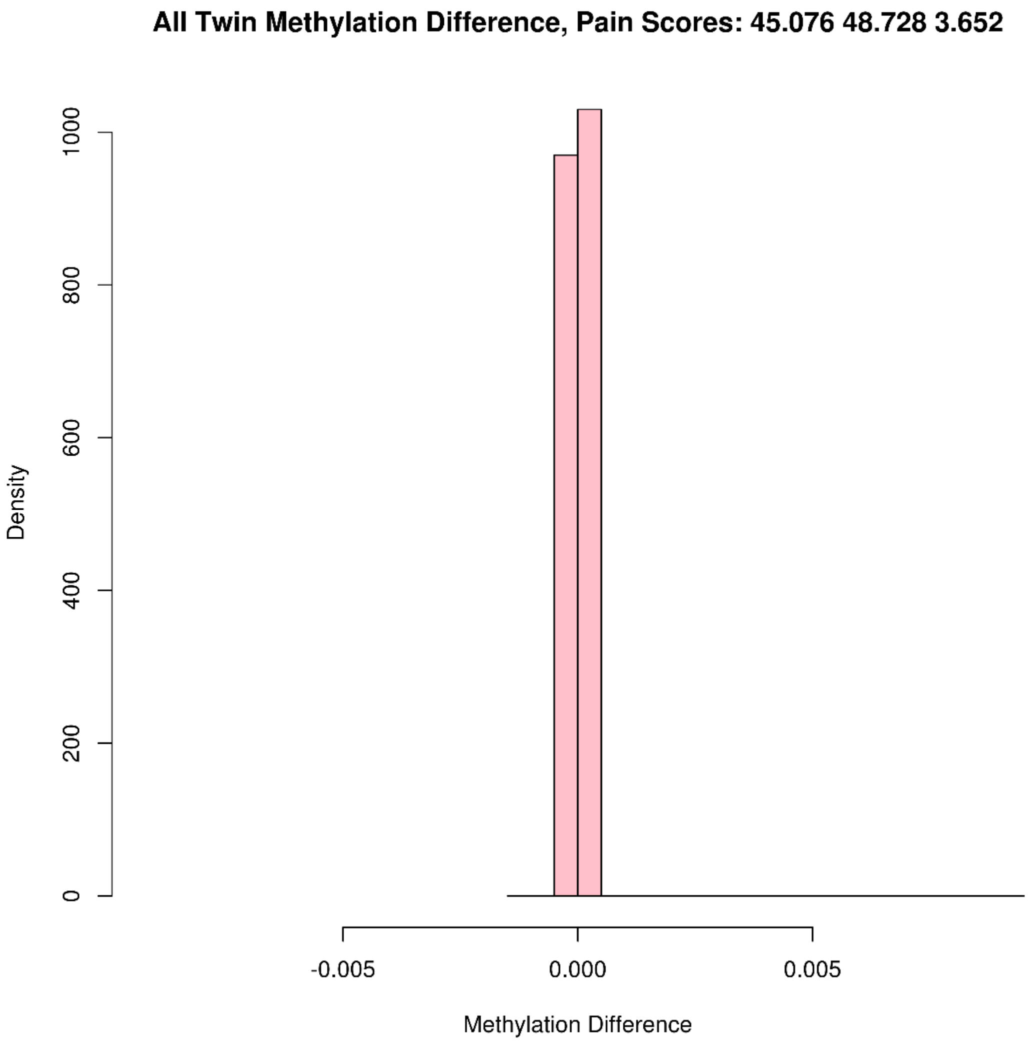

Figure S3: Mean methylation profiles between higher and lower pain temperature group in 25 MZ twin pairs

Table S1 Number of significant DMCs, genes recognized by Ensemble by applying WFMM δ=4x10^{-5} and qvalue=1.01, difference=0.07 on 25 monozygotic twin pairs with different pain sensitivity temperature for each chromosome.

Chrom	WFMM δ=4x10^{-5}, Number of DMRs	methylKit, qvalue=1.01, difference=0.07, Number of DMRs	WFMM δ=4x10^{-5}, Number of significant genes from Ensemble	methylKit, qvalue=1.01, difference=0.07, Number of significant genes from Ensemble
Chr1	53	59	21	35
Chr2	23	28	9	23
Chr3	3	3	1	2
Chr4	25	17	10	9
Chr5	10	16	3	8
Chr6	40	21	11	8
Chr7	31	25	19	15
Chr8	36	33	11	12
Chr9	22	21	5	7
Chr10	50	40	11	9
Chr11	20	20	9	11
Chr12	0	15	0	9
Chr13	0	6	0	2
Chr14	7	13	4	4
Chr15	8	11	1	3
Chr16	78	54	21	25
Chr17	27	24	10	13
Chr18	11	15	5	7
Chr19	12	45	4	21
Chr20	10	30	5	11
Chr21	9	20	4	10
Chr22	19	30	3	9
Total	494	546	167	253
